# Supplementary material for: Pathogen invasion-dependent tissue reservoirs and plasmid-encoded antibiotic degradation boost plasmid spread in the gut
Source: eLife. 2021 Dec 7;10:e69744. doi: 10.7554/eLife.69744 (PMC8651294; doi:10.7554/eLife.69744)
Supplement: Transparent reporting form [file elife-69744-transrepform1.docx]

***eLife’s* transparent reporting form**

We encourage authors to provide detailed information *within their submission* to facilitate the interpretation and replication of experiments. Authors can upload supporting documentation to indicate the use of appropriate reporting guidelines for health-related research (see [EQUATOR Network](http://www.equator-network.org/%20)), life science research (see the [BioSharing Information Resource](https://biosharing.org/)), or the [ARRIVE guidelines](http://www.plosbiology.org/article/info:doi/10.1371/journal.pbio.1000412) for reporting work involving animal research. Where applicable, authors should refer to any relevant reporting standards documents in this form.

If you have any questions, please consult our Journal Policies and/or contact us: [editorial@elifesciences.org](mailto:editorial@elifesciences.org).

**Sample-size estimation**

- You should state whether an appropriate sample size was computed when the study was being designed
- You should state the statistical method of sample size computation and any required assumptions
- If no explicit power analysis was used, you should describe how you decided what sample (replicate) size (number) to use

Please outline where this information can be found within the submission (e.g., sections or figure legends), or explain why this information doesn’t apply to your submission:

Sample size was not predetermined. Since large differences in phenotypes were expected, 5-10 mice were generally used, with mice randomly assigned to treatment group. In key experiments, up to 13 mice were used to ensure claims were accurate and encompass a larger range of possible biological variation. Sample size and group allocation methods are described in the material and methods section “infection experiments”.

**Replicates**

- You should report how often each experiment was performed
- You should include a definition of biological versus technical replication
- The data obtained should be provided and sufficient information should be provided to indicate the number of independent biological and/or technical replicates
- If you encountered any outliers, you should describe how these were handled
- Criteria for exclusion/inclusion of data should be clearly stated
- High-throughput sequence data should be uploaded before submission, with a private link for reviewers provided (these are available from both GEO and ArrayExpress)

Please outline where this information can be found within the submission (e.g., sections or figure legends), or explain why this information doesn’t apply to your submission:

The number of independent biological replicates are indicated in the figure captions. Since data is derived from mice, there are no technical replicates, only biological replicates. Each experiment was repeated a minimum of 2 times. To ensure experimental independence, mice were caged individually for the maximum duration that this ethically feasible (10 days). Otherwise, mice were caged as pairs. This information is detailed in the material and methods section “infection experiments”. There were two criteria for exclusion of data: 1) failure of S.Tm to colonize systemic sites after i.p. injection (which occurs in <5-10% of mice, likely due to technical reasons) which meant that it was impossible to analyse reseeding, 2) disease state of mice (clearly defined severity endpoint required premature euthanasia and subsequent exclusion of mice in a few cases). If S.Tm was detected in the systemic sites (e.g. spleen; positive control for colonization of systemic sites) and disease severity was maintained below a clearly defined threshold, mice were not excluded. For fitting of data to the mathematical model, experimental data was included in cases where the assumptions of our model were true. This may cause an overestimation of the migration rate, but we do not expect the relative contribution of rates to change. This is detailed in the supplementary information section “parameter estimation in the mathematical model”.

**Statistical reporting**

- Statistical analysis methods should be described and justified
- Raw data should be presented in figures whenever informative to do so (typically when N per group is less than 10)
- For each experiment, you should identify the statistical tests used, exact values of N, definitions of center, methods of multiple test correction, and dispersion and precision measures (e.g., mean, median, SD, SEM, confidence intervals; and, for the major substantive results, a measure of effect size (e.g., Pearson's r, Cohen's d)
- Report exact p-values wherever possible alongside the summary statistics and 95% confidence intervals. These should be reported for all key questions and not only when the p-value is less than 0.05.

Please outline where this information can be found within the submission (e.g., sections or figure legends), or explain why this information doesn’t apply to your submission:

Each statistical test performed is described in the figure legends. Exact p values are not presented in the manuscript but are included in the source data (see below). Non-parametric testing was used for mouse data since normality cannot be assumed. All data points are presented as scatter plots with bars or lines indicating the median. For fitting of the mathematical model to the experimental values, we used an Approximate Bayesian Computation (ABC) approach (the reference Marjoram et al. 2003, PNAS describes this in detail). This is detailed in the supplementary information.

(For large datasets, or papers with a very large number of statistical tests, you may upload a single table file with tests, Ns, etc., with reference to sections in the manuscript.)

**Group allocation**

- Indicate how samples were allocated into experimental groups (in the case of clinical studies, please specify allocation to treatment method); if randomization was used, please also state if restricted randomization was applied
- Indicate if masking was used during group allocation, data collection and/or data analysis

Please outline where this information can be found within the submission (e.g., sections or figure legends), or explain why this information doesn’t apply to your submission:

Mice were randomly assigned to treatment group and experiments were not blinded. This is explained by the objective nature of colony counting from selective plating and qPCR analysis used for the experimental results.

**Additional data files (“source data”)**

- We encourage you to upload relevant additional data files, such as numerical data that are represented as a graph in a figure, or as a summary table
- Where provided, these should be in the most useful format, and they can be uploaded as “Source data” files linked to a main figure or table
- Include model definition files including the full list of parameters used
- Include code used for data analysis (e.g., R, MatLab)
- Avoid stating that data files are “available upon request”

Please indicate the figures or tables for which source data files have been provided:

Source data is provided for all figures that contain graphs derived from experimental data as excel sheets. P values are indicated in the excel sheets. All parameters used for the mathematical model are listed in the supplementary information (table S1). All R-code needed to simulate the stochastic model, estimate the most likely parameters from the experimental data, and plot the results, is included in the Github repository https://github.com/JSHuisman/Recorder . The experimental source data sheets are also found at that Github repository.
